# Changes of dysfunctional lens index before and after implantable collamer lens V4c implantation in patients with moderate-to-high myopia

**DOI:** 10.1007/s10792-023-02812-0

**Published:** 2023-07-22

**Authors:** Weifang Cao, Suhua Zhang, Qian Liu, Jing Zhou, Xiaoyong Yuan

**Affiliations:** 1 Clinical College of Ophthalmology, Tianjin Medical University, Tianjin, 300070 China; 2 Tianjin Key Laboratory of Ophthalmology and Visual Science, Tianjin Eye Institute, Tianjin Eye Hospital, Tianjin, 300020 China; 3 Department of Cataract, Shanxi Eye Hospital, Taiyuan, 030002 Shanxi Province China

**Keywords:** Phakic intraocular lens, Moderate-to-high myopia, Refractive surgery, Dysfunctional lens index

## Abstract

**Background:**

Dysfunctional lens index (DLI) changing is rarely reported after implantable collamer lens (ICL) implantation. In the current research, we hope to investigate the changes of DLI by ray-tracing aberrometry before and after implantation of the posterior chamber phakic implantable collamer lens with a central artificial hole for patients with moderate-to-high myopia.

**Methods:**

This retrospective, observational case series included 206 eyes of 104 patients with moderate-to-high myopia who underwent ICL V4c implantation. Data were collected on ocular indicators preoperatively and at 1 day, 1, 3, and 6 months postoperatively. The i-Trace Visual Functional Analyzer was used to assess the DLI measurement.

**Results:**

The overall values of safety index and efficacy index were both more than 1. Preoperatively, the mean spherical equivalent (SE) of included 206 eyes was − 10.77 ± 3.46 diopter (D). Then at 1-day postoperation, the mean SE was − 0.22 ± 0.55 D, and barely changed from 1 day to 6 months postoperatively. Although the endothelial parameters had no significant differences between preoperation and postoperation, the mean loss of endothelial cells was 0.74 ± 0.98% at 6 months. Regarding the vault, there was a significant difference between each time of follow-up (*P* < 0.001). The mean of the vault decreased 109.6 ± 13.5 µm from 1-day post-op to 6 months post-op. The DLI values were 3.70, 9.26, 10.00, and 9.68 at baseline, 1, 3, and 6 months, respectively (*P* < 0.001), but no significant differences were found between 1, 3, and 6 months postoperatively (*P* > 0.05). The preoperative lnDLI showed a significant positive linear correlation (*r* = 0.621, *P* < 0.001) with the preoperative spherical equivalent (SE). The lnDLI was negatively correlated with the axial length (*r* = − 0.462, *P* < 0.001), corneal thickness (*r* = − 0.207, *P* = 0.003), preoperative LogMAR UDVA (*r* = − 0.189, *P* = 0.006), and preoperative LogMAR CDVA (*r* = − 0.306, *P* < 0.001).

**Conclusions:**

The postoperative refractive parameters were confirmed excellent in efficacy, predictability, and stability in half a year. The DLI was significantly improved after the ICL V4c implantation in patients with moderate-to-high myopia and showed good stability during the follow-up periods. The DLI deserves a more comprehensive understanding and application in clinical services.

## Background

The number of individuals with myopia has increased over the past years, especially in East and Southeast Asia, where it is seen in up to 90% of adolescents, a vast increase compared with an incidence of maximum 30% in adolescents 60 years ago [1, 2]. Phakic intraocular lenses (PIOL) have been widely used for the correction of various degrees of myopic or combined with astigmatism [3]. One of the most widely-used types of PIOL is the implantable collamer lens (ICL; STAAR Surgical Inc., Monrovia, CA, USA). Its model V4c with a 0.36-mm central hole has been gradually accepted for making iridectomies or iridotomies unnecessary and allowing for adequate aqueous flow physiologically through the anterior segment [4, 5]. Compared with corneal refractive surgery such as small incision lenticule extraction (SMILE) and femtosecond laser-assisted *in situ* keratomileusis (FS-LASIK), the advantages of ICL implantation include equally matched efficacy, safety, stability, and accuracy [6], but better on the visual acuity of postoperative recovery [7]. Particularly, the ICL V4c was well-received because its advantages of wide range, stability to the biomechanical structure of the cornea, and less risk of complications such as dry eye and reversibility.

The dysfunctional lens index (DLI) measured by ray-tracing aberrometry was an objective lens performance metric and had a positive correlation with the LOCS III nuclear opalescence (NO) score, Scheimpflug-measured lens nuclear density value, and corrected distance visual acuity (CDVA) in patients with cataract [8]. Furthermore, the DLI provides an indicator to evaluate the grade of nuclear cataract, crystalline lens status, and the monitoring of disease progression. It has been used to help guide ophthalmologists making the decision whether cataracts should be treated by surgery [9]. However, to the best of our knowledge, few investigations have been performed to observe the changes of DLI in myopic with ICL V4c implantation.

Therefore, the current study focused on the relationship between DLI and the implantation of ICL V4c in patients with moderate-to-high myopia and devoted to discovering a more comprehensive clinical significance of this objective metric.

## Methods

This study was approved by the Institutional Review Board (IRB) of Shanxi Eye Hospital and followed the tenets of the Declaration of Helsinki. After a detailed and careful communication, written informed consent was obtained from each participant.

This retrospective observational case series was conducted with patients who were consecutively enrolled from March 2018 to May 2019 at Shanxi Eye Hospital (Taiyuan City, Shanxi Province, China). All of them had been suffered with moderate-to-high myopia and had a strong desire to take the glasses off.

Inclusion criteria were as follows: (1) age greater than 18 years but lower than 45 years; (2) endothelial cell count ≥ 2000 cells/mm^2^ and inner anterior chamber depth ≥ 2.8 mm; (3) stable refraction (change in mean refractive spherical equivalent ≤ 0.5 D) for a minimum period of 2 years; (4) stop using soft contact lens at least 2 weeks; and (5) stop using rigid gas permeable contact lens at least 4 weeks. Patient had a general disease such as autoimmune disease or diabetes which will affect the operation, with previously ocular surgery, or comorbidities including trauma, lens opacity, glaucoma, uveitis, diabetic retinopathy, and corneal ectasia were excluded from the study.

Preoperatively, all participants underwent a comprehensive ophthalmic examination, including slit-lamp biomicroscopy and dilated fundus evaluation. Biometric parameters including corneal thickness (CT), the horizontal white-to-white (WTW), inner anterior chamber depth (ACD), and keratometric values were obtained by Orbscan II (Bausch & Lomb Surgical, Inc.) and Pentacam HR (Oculus Optikgerate, Wetzlar, Germany). Axial length (AL) was also measured by IOL Master 500 (Carl Zeiss AG, Germany).

The following parameters were compared preoperatively and at day 1, 1, 3, and 6 months postoperative visits: uncorrected distance visual acuity (UDVA), corrected distance visual acuity (CDVA, recorded in LogMAR units), manifest refraction spherical equivalent (MRSE), intraocular pressure (IOP) using a noncontact tonometry Canon Full Auto Tonometer TX-F (Canon, Inc., Tokyo, Japan), endothelial cell density (ECD) by SP-3000P (Topcon Corporation, Kyoto, Japan), the vault (distance between the posterior surface of the ICL and the anterior lens capsule) measured by an Optovue Angio OCT (Optovue Inc., Fremont, California, USA), and DLI by i-Trace Visual Function Analyzer (Tracey Technologies, Houston, TX).

The safety index (SI) was defined as postoperative CDVA over preoperative CDVA, and the efficacy index (EI) was defined as the postoperative UDVA over preoperative CDVA.

### Implantable collamer lens

All participants underwent ICL V4c implantation for myopic correction by the same senior surgeon, and most of them had a minimum follow-up period of half a year. The ICL V4c (Visian ICL™, STAAR Surgical Company, Monrovia, CA, USA) is a phakic lens designed with a 360-µm central hole made from collamer (STAAR Surgical AG proprietary), which is a flexible, hydrophilic, and biocompatible material. It has a plate-haptic design and a central convex/concave optical zone, allowing for posterior chamber injection with support on the ciliary sulcus through a microscopic incision of 3.2 mm or smaller. The EVO ICL is indicated for phakic patients with a spherical equivalent ranging from −0.50 to −18.0 D and up to 6.00 D cylindrical refraction [10]. In the current study, IOL power and size were performed based on WTW, ACD, and MRSE by an online system (STAAR Surgical Co., Monrovia, CA, USA).

### Dysfunctional lens index measurement

The i-Trace Visual Function Analyzer integrates an aberrometer with a corneal topographer. The aberrometer used the ray-tracing principle by sequentially project 256 near-infrared laser beams into the eye within a specific scanning pattern and parameter detection takes less than 200 ms. Topographies were captured using a Placido-based corneal topographer mounted on the same device. The corneal aberrations were obtained by anterior topography data, and the internal aberrations were obtained by subtracting the corneal wavefront aberrations from the entire eye using the built-in program. The DLI has always been considered an objective lens performance parameter provided by this wavefront sensor and is calculated based on the internal higher-order aberrations (HOAs), pupil size, and contrast sensitivity data [11].

Three measurements of each eye were performed in a darkroom. Before each measurement, patients were asked to blink a couple of times. A measurement was taken in a 3-mm diameter zone set by the device, and the DLI was registered for each eye included in the study. This objective index ranks the overall lens performance from 0 (very poor) to 10 (excellent) points. The values of natural logarithm (ln)-transformed were applied to obtain a normal distribution for the preoperative DLI values, because it showed a left-skewed distribution.

**Fig. 1:**
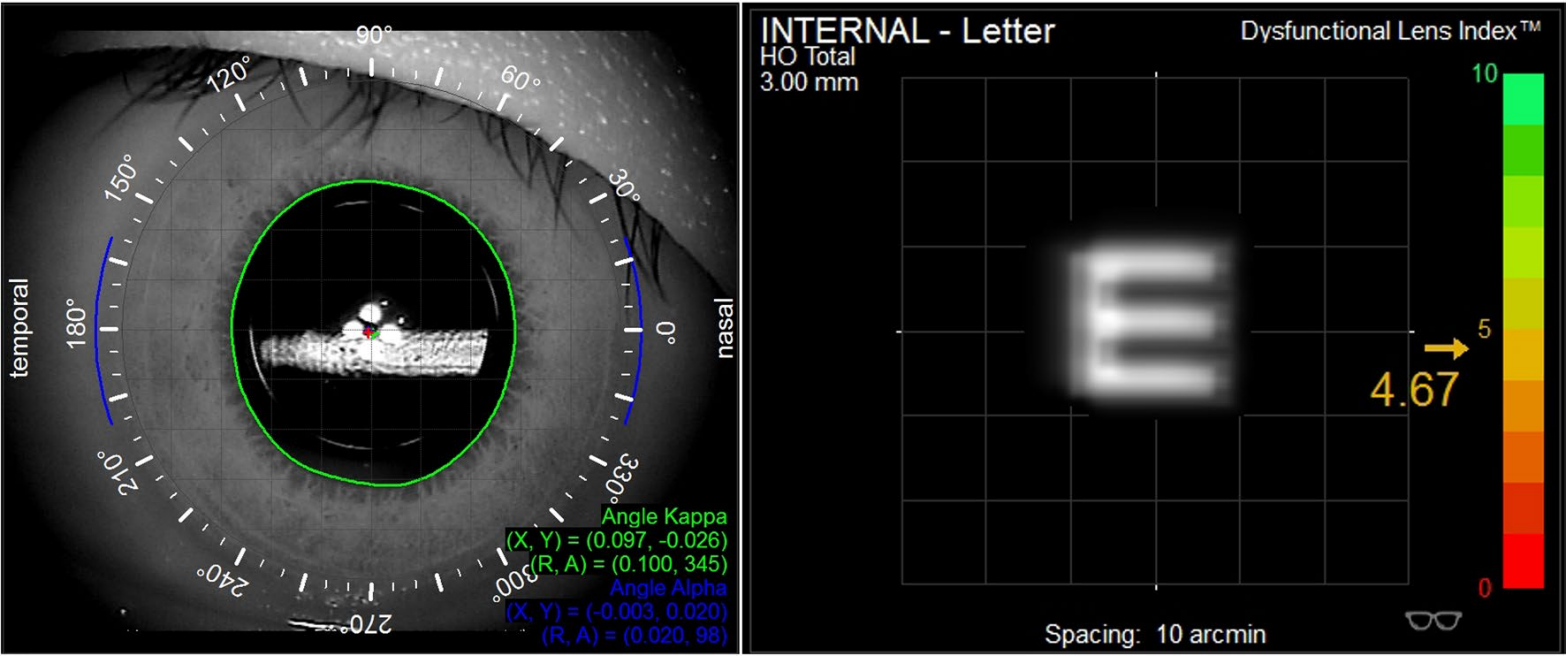
The i-Trace scan postoperatively containing ICL artifacts would influence the accuracy and authenticity of the measurement

An experienced rater (WC) collected all the databases for every patient and reviewed the i-Trace scans not only to regard the centration of the measurement on the visual axis of the eye, but also to delete the images containing ICL artifacts which would influence the accuracy and authenticity of the measurement (Fig. 1). Then, the best-quality scan for each eye was selected for further statistical analysis.

### Surgical technique

The same surgeon (Dr. SZ) performed all surgeries to guarantee the validity of the study. Preoperatively, the operative eye was given 0.5% levofloxacin eye drops (Santen Pharmaceutical Co., Ltd., Osaka, Japan) for 4 times/day within 48 h. At 30 min before surgery, compound tropicamide eye drops were used for mydriasis. The axis of astigmatism was marked under the slit lamp before surgery at normal pupil size. Conventional disinfection and draping were conducted. The main incision was made at the steepest meridian of the cornea. ICL V4c was implanted via a 3-mm clear corneal incision using an injector cartridge. Then, a small amount of viscoelastic surgical agent (1% sodium hyaluronate) was injected into the anterior chamber above the front surface of the ICL. After the four tentacles were placed in the posterior chamber, the viscoelastic surgical agent was washed away using balanced salt solution. Antibiotic eye drops, nonsteroidal anti-inflammatory eye drops, and steroidal eye drops were given to the operative eyes postoperatively.

### Data and statistical analysis

All statistical analysis was performed using SPSS version 26.0 (SPSS Inc., Chicago, Illinois, USA). Kolmogorov–Smirnov test was used to check the normal distribution of each variable. Normally distributed parameters were expressed as mean ± standard division (SD), while non-normally distributed parameters were expressed as median (interquartile range). One-way repeated-measures analysis of variance was used to compare the changes of indicators pre- and post-treatment. Greenhouse–Geisser method was used for correction and pairwise comparison between post hoc test groups by Bonferroni method. Friedman test and Wilcoxon rank-sum test were used to compare the collected parameters with non-normal distributions. Pearson/Spearman analysis was used for correlation analysis. A P value less than 0.05 was considered statistically significant.

## Results

### Characteristics of the patients

A total of 206 eyes (103 right eyes and 103 left eyes) of 104 patients (two unilateral and 102 bilateral) were recruited, consisting of 36 males and 68 females. The mean preoperative spherical diopter of the ICL V4c implanted in all participants was − 11.86 ± 3.25 D (range − 18.0 D–− 3.5 D), and the mean preoperative cylindrical diopter of the ICL V4c was 0.81 ± 1.08 D (range 0 D–4.0 D). Toric ICL V4c was implanted in 85 (41.3%) eyes. There are four sizes were implanted, including 12.1 mm (43 eyes, 20.9%), 12.6 mm (99 eyes, 48.1%), 13.2 mm (60 eyes, 29.1%), and 13.7 mm (four eyes, 1.9%). Table 1 shows an overview of the preoperative characteristics of the included patients.

**Table 1:** Preoperative patient demographics (*n* = 206)

Characteristic	Mean ± SD	Range
Age (years)	26.11 ± 5.38	[18, 42]
AL-IOL Master 500 (mm)	27.41 ± 1.61	[24.19, 32.88]
ACD-Pentacam HR (mm)	3.26 ± 0.25	[2.80, 3.88]
K-flat-Pentacam HR (D)	43.12 ± 1.54	[39.40, 48.20]
K-steep-Pentacam HR (D)	44.67 ± 1.71	[41.20, 50.20]
CT-Pentacam HR (µm)	525.1 ± 31.8	[444, 593]
WTW-Pentacam HR (mm)	11.56 ± 0.43	[10.40, 12.60]
ACD-Orbscan II (mm)	3.12 ± 0.25	[2.81. 3.77]
K-flat-Orbscan II (D)	43.38 ± 1.63	[39.50, 48.70]
K-steep-Orbscan II (D)	44.92 ± 1.76	[41.30, 50.50]
CT-Orbscan II (µm)	530.9 ± 44.26	[420, 629]
WTW-Orbscan II (mm)	11.49 ± 0.39	[10.70, 12.90]
WTW-IOL Master 500 (mm)	11.69 ± 0.44	[10.60, 13.00]

*AL* Axial length; *ACD* Inner anterior chamber depth; *K* Keratometric; *CT* Corneal thickness; and *WTW* White-to-white distance

### Visual acuity and refraction

**Fig. 2:**
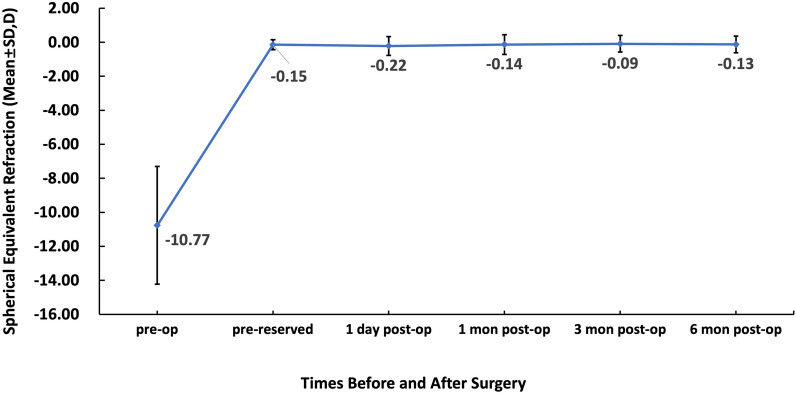
The spherical equivalent (SE) outcomes preoperatively, reserved before operation and at 1-day, 1, 3, and 6 months postoperatively

**Fig. 3:**
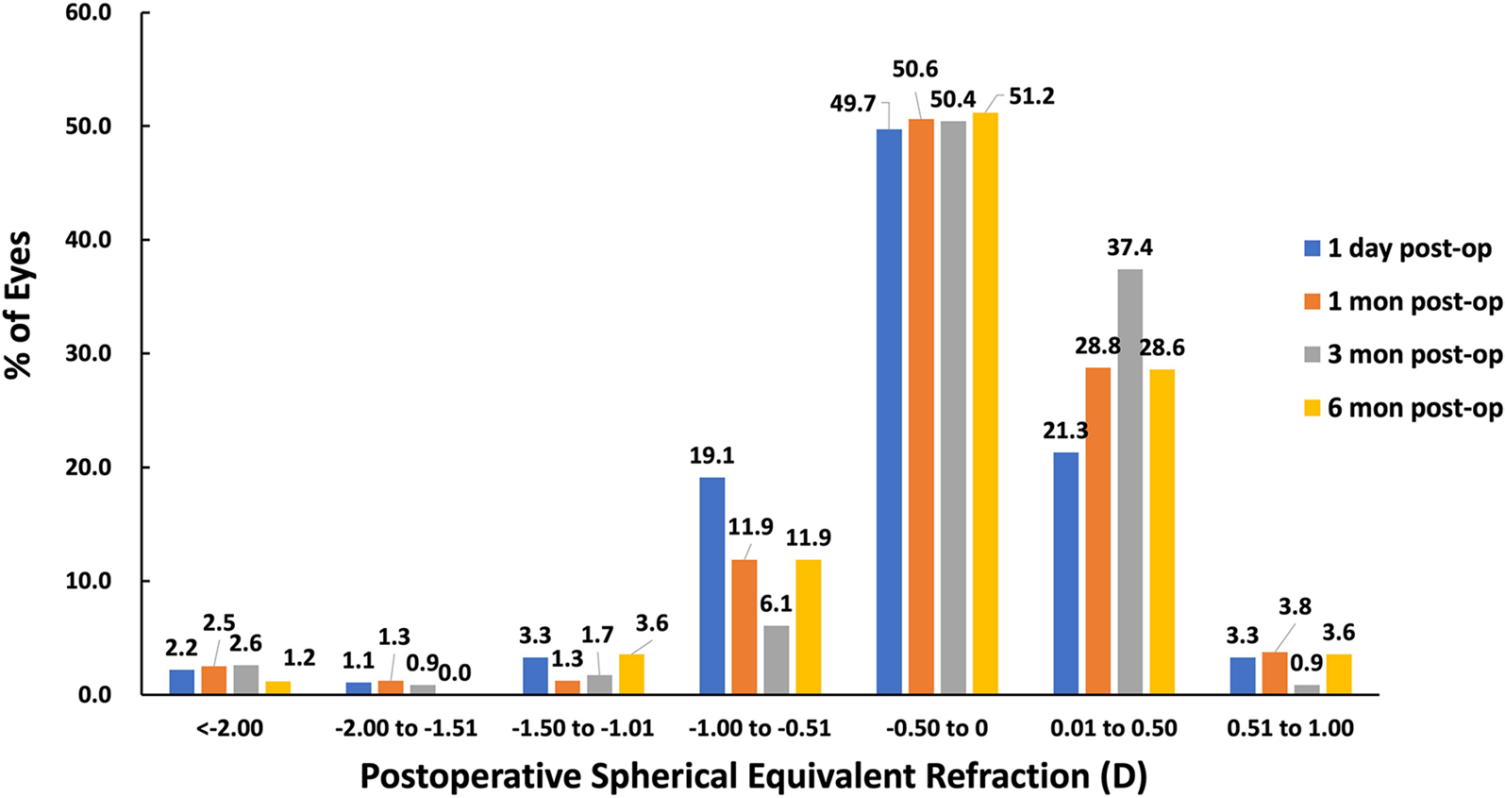
The spherical equivalent (SE) refractive accuracy at 1 day and 1, 3, and 6 months postoperatively

Preoperatively, the mean spherical equivalent (SE) of the 206 eyes was − 10.77 ± 3.46 D (range: − 3.25 D–− 20.62 D), and the preoperative reserved SE was − 0.15 ± 0.30 D (range: − 2.06 D–0.35 D). Then at 1-day postoperation, the mean SE of the 206 eyes was − 0.22 ± 0.55 D (range: − 3.25 D–0.75 D), and the SE refraction barely changed from 1 day to 6 months (Fig. 2). At 1 day and 1, 3, and 6 months, 71.0%, 79.4%, 87.8%, and 79.8% of the eyes had an SE refraction within ± 0.5 D, respectively, and 93.4%, 95.1%, 94.8%, and 95.3% within ± 1.0 D, respectively (Fig. 3). Table 2 shows the refractive outcomes of the eyes before and after the ICL V4c implantation at 1 day and 1, 3, and 6 months.

**Table 2:** The clinical parameters of the eyes before and after the implantable collamer lens V4c implantation (*n* = 206)

Characteristic	Preoperative (Mean ± SD)	Postoperative (Mean ± SD)				P1	P2
		1 day	1 months	3 months	6 months		
LogMAR UDVA	1.26 ± 0.37	− 0.02 ± 0.10	− 0.02 ± 0.08	− 0.03 ± 0.09	− 0.01 ± 0.09	< 0.001	0.551
LogMAR CDVA	0.03 ± 0.09	− 0.05 ± 0.07	− 0.06 ± 0.08	− 0.06 ± 0.07	− 0.06 ± 0.07	< 0.001	0.371
SI	NA	1.18 ± 0.26	1.19 ± 0.23	1.18 ± 0.25	1.19 ± 0.27		0.498
EI	NA	1.10 ± 0.23	1.09 ± 0.21	1.11 ± 0.22	1.08 ± 0.22		0.479
Sphere (D)	− 10.09 ± 3.35	0.02 ± 0.47	0.01 ± 0.44	0.06 ± 0.41	0.04 ± 0.50	< 0.001	0.884
Cylinder (D)	− 1.34 ± 1.03	− 0.47 ± 0.72	− 0.30 ± 0.72	− 0.30 ± 0.68	− 0.33 ± 0.66	< 0.001	0.318
SE (D)	− 10.77 ± 3.46	− 0.22 ± 0.55	− 0.14 ± 0.57	− 0.09 ± 0.49	− 0.13 ± 0.49	< 0.001	0.426
IOP	15.60 ± 2.69	16.92 ± 3.47	16.18 ± 3.30	15.78 ± 2.44	15.42 ± 2.71	0.013	0.019
ECD	2762.60 ± 259.27	NA	2742.10 ± 288.68	2728.64 ± 281.72	2739.30 ± 301.72	0.313	0.553
ECV	35.97 ± 8.30	NA	34.37 ± 5.43	34.17 ± 6.17	34.14 ± 5.07	0.473	0.506
EHE	55.01 ± 13.32	NA	55.40 ± 11.87	55.60 ± 11.24	54.94 ± 11.95	0.976	0.979
Vault (µm)	NA	663.9 ± 278.5	606.3 ± 262.1	561.8 ± 237.0	554.3 ± 218.3		< 0.001

*LogMAR* The logarithm of the minimal angle of resolution; *UDVA* Uncorrected distance visual acuity; *CDVA* Corrected distance visual acuity; *SI* Safety index; *EI* Efficacy index; *SE* Spherical equivalent; *IOP* Intraocular pressure; *ECD* Endothelial cell density; *ECV* Endothelial variable coefficient for size; *EHE* Endothelial hexagonality; *NA* Not applicable; *P1* *P* value between preoperative and postoperative follow-up visits; and *P2* *P* value between postoperative visits

No significant differences were observed in SE, sphere diopter, cylinder diopter, LogMAR UDVA, LogMAR CDVA, SI, and EI between the postoperative follow-up visits (*P* > 0.05). However, there were significant statistical differences between pre- and post-periods regarding LogMAR UDVA or CDVA, refractive sphere, refractive cylinder, and SE (*P* < 0.01).

### Security parameters

The security parameters of every period are also summarized in Table 2. No significant differences were observed about endothelial cell density, variable coefficient for size, and hexagonality between preoperatively and 1, 3, and 6 months postoperatively (*P* > 0.05).

Regarding the IOP, there was a significant statistical difference between 1-day postoperatively and all other visits, including preoperation (*P* = 0.013).

For vault values, distance from the posterior surface of the ICL to the anterior lens capsule, there was a significant difference between every period of follow-up time (*P* < 0.001). The mean of the vault decreased 109.6 ± 13.5 µm (95% confidence interval for difference 72.9–146.3 µm) from 1-day postoperatively to 6 months postoperatively.

### Dysfunctional lens index

**Fig. 4:**
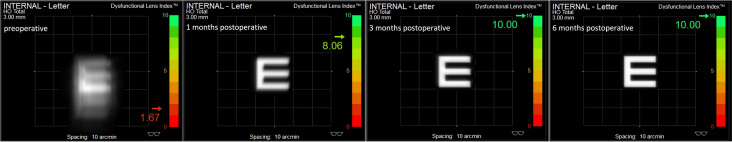
The i-Trace scans of one patient’s left eye preoperation and at 1, 3, and 6 months postoperatively

**Fig. 5:**
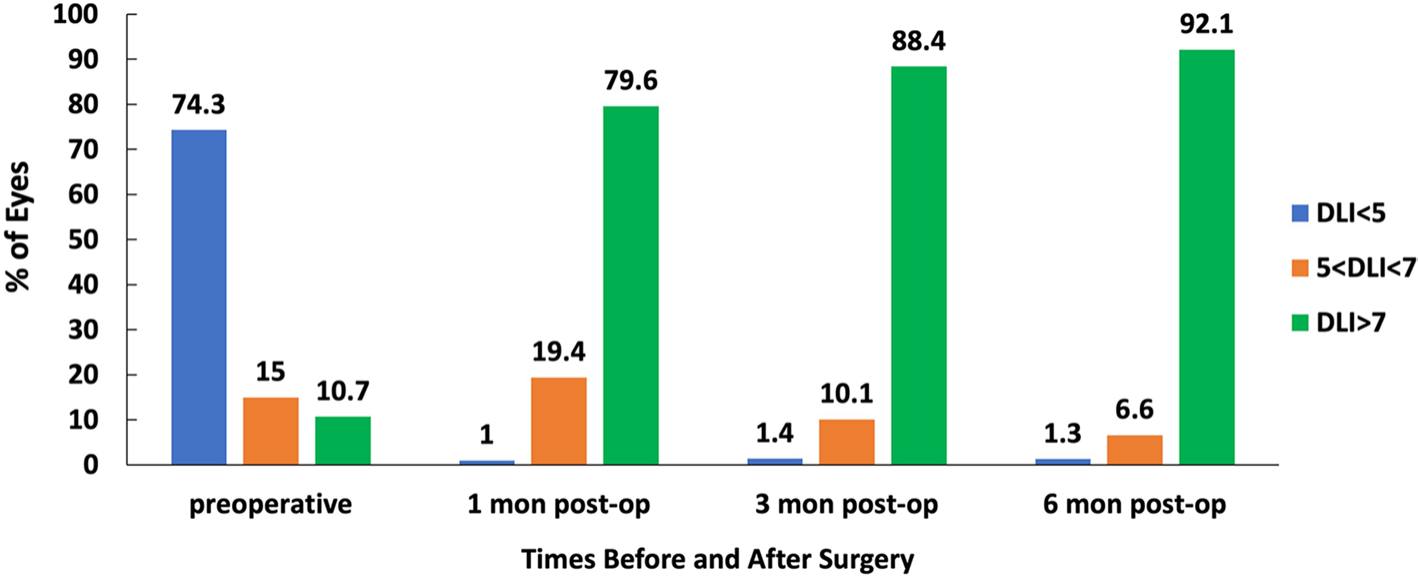
The distribution of DLI measured by i-Trace preoperatively and at 1, 3, and 6 months after implantation of posterior chamber phakic implantable collamer lens with a central hole (ICL V4c)

The DLI measured by i-Trace was 3.70 (2.56, 5.04), 9.26 (7.37, 10.00), 10.00 (8.06, 10.0), and 9.68 (8.24, 10.00) at baseline and 1, 3, and 6 postoperative months, respectively (*P* < 0.001), while no significant differences were found between 1, 3, and 6 months (*P* = 0.736, 0.469, and 0.700, respectively). The DLI was significantly improved after the ICL V4c implantation compared with those before surgery in patients with moderate-to-high myopia and showed good stability during the follow-up period (Fig. 4). The DLI value greater than 7 was considered normal, while it less than 5 was considered abnormal and even required treatment. Preoperation and at 1, 3, and 6 months postoperatively, the DLI values greater than 7 were 10.7%, 79.6%, 88.4%, and 92.1%, respectively (Fig. 5).

The preoperative lnDLI showed a high-positive linear correlation (*r* = 0.621, *P* < 0.001) with the preoperative spherical equivalent (SE). Respectively, the lnDLI showed a positive linear correlation with the preoperative sphere diopter (*r* = 0.618, *P* < 0.001) and the preoperative cylinder diopter (*r* = 0.159, *P* = 0.022). The lnDLI was negatively correlated with the axial length (*r* = − 0.462, *P* < 0.001) and corneal thickness (*r* = − 0.207, *P* = 0.003). However, there was no significant correlation between the lnDLI and the horizontal white-to-white anterior chamber depth, keratometric values, and age (*r* = 0.108, − 0.054, − 0.023, and 0.040, respectively, *P* > 0.05). The lnDLI was negatively correlated with preoperative LogMAR UDVA (*r* = − 0.189, *P* = 0.006) and preoperative LogMAR CDVA (*r* = − 0.306, *P* < 0.001).

## Discussion

The ICL V4c was first implanted by Kimiya Shimizu in 2007 [12], and the number of ICL surgeries has been rapidly increased due to its advantages. Even in middle-aged patients, aged 45–65 years, during the 2.2 follow-up years, hole ICL implantation offered favorable outcomes in all measures of safety, efficacy, predictability, and stability [13]. However, the conventional ICL has two drawbacks. One is the need for iridectomy, and the other is the progression of anterior subcapsular cataract (ASC) after ICL implantation. Especially the incidence of ASC ranged from 1.1 to 5.9%, and the probability of requiring cataract surgery for vision loss ranged from 0 to 1.8% [14]. In an attempt to overcome complications, the ICL V4c incorporated a 0.36-mm central port, thus making iridectomies or iridotomies unnecessary and allowing for adequate aqueous flow maintaining the normal physiology of the anterior segment to avoid ASC [3].

In the present study, the values of SI and EI were both greater than 1. The postoperative visual acuity and refractive parameters were also confirmed excellent in efficacy, predictability, and stability in half a year observation [14]. Although the endothelial parameters had no significant differences during preoperation and postoperation, the mean rate of endothelial cell loss was 0.74 ± 0.98% at 6 months postoperation, which was higher than the natural physiological loss of endothelial cells approximately to be 0.6 ± 0.5% per year in human [15]. Regarding another parameter of safety, the mean of IOP 1-day postoperatively increased 1.32 ± 0.45 mmHg than preoperation. The possible cause was a little amount of viscoelastic agent residue during the operation. Moreover, the most challenging segment in ICL implantation is the accurate prediction of vaulting [14]. Still, if conditions permitted, the intraoperative OCT system could be used to predict the vaulting value [16]. A previous study showed that ambient light conditions affected vaulting value [17]. Therefore, we performed all measurements under the same indoor light. Multiple studies have suggested 250–750 μm as normal vaulting [18, 19]. In this study, the mean of the vault had been maintained above 500 μm. However, the mean value decreased 109.6 ± 13.5 µm at 6 months follow-up visit, which suggested that a longer follow-up is necessary.

Currently, the ray-tracing aberrometer (i-Trace) is widely used due to its advantages over Hartmann–Shack and other types of wavefront sensors [20]. This i-Trace Visual Function Analyzer projects 256 near-infrared laser beams in a programmable scan pattern across the pupil in succession and detects the retinal location of reflection. Based on these measurements, it calculates the retinal point spread function and subsequently provides several parameters, including the DLI and ocular HOAs. Wavefront analysis objectively evaluates the visual deterioration by quantifying the HOAs of the ocular optical system. The previous reports demonstrated a high correlation between the DLI and the Lens Opacities Classification System III (LOCS III) score, which was a subjective metric and Scheimpflug-based average lens density in a three-dimensional cylindrical template, which was an objective metric, as well as LogMAR CDVA [8, 9]. Both the quantification parameters derived from Scheimpflug densitometry and the functional status of the crystalline lens based on ray-tracing aberrometry in eyes with mild nuclear cataract might help in preoperative counseling and patient education in clinical practice [21]. Furthermore, a previous study reported the DLI was useful for the phacoemulsification dynamics prediction in age-related nuclear cataract, even better than Scheimpflug-derived average density and LOCS III nuclear opalescence [11]. Herein, our findings confirmed that the DLI was significantly improved after the ICL V4c implantation in moderate-to-high myopic eyes, and showed a good stability during the follow-up periods.

To our knowledge, this study is the first to establish the relationship between DLI and ICL implantation outcomes in eyes with myopia. Although the DLI is a dysfunctional lens index as its name implies, and some previous studies have used DLI to observe changes in lens density as an objective indicator to guide cataract surgery. However, in the process of ICL surgery, we found that the preoperative and postoperative DLI values of patients with high myopia were significantly different, which was statistically significant. The DLI value may indicate not only the functional status of the lens nucleus, but also the refractive state and visual quantification of the inner optical system. Another advantage of the DLI is their presentation on a continuous scale, allowing a better and more precise assessment for clinical and research purposes. We hope that in the process of clinical work, we are not limited to the lens' function, but to explore its new meaning.

In this study, the limitation includes the small number of cases and the absence of age-matched controls. A study with a larger number of patients with normal age-matched controls would be appropriate. Secondly, our findings might have potential selection bias, because we did not include both normal and low myopia patients. Thirdly, the pupil size might be another important factor which has correlation with optical quality change and DLI value. The measurement was taken in a 3-mm diameter zone, and the DLI was registered for each eye in our study. Further comparison of the pupil size and the DLI value for optical quality needs a long-term follow-up and further research.

## Conclusion

Our results suggest that the DLI is a valuable parameter in monitoring the refractive state and visual quantification of the optical system. Although there is no consensus on this parameter, it may deserve better evaluation and clinical application for patients with myopia.

## Data Availability

The datasets used during the current study are available from the corresponding author on reasonable request.

## Abbreviations

LogMAR: The logarithm of the minimal angle of resolution
UDVA: Uncorrected distance visual acuity
CDVA: Corrected distance visual acuity
SI: Safety index
EI: Efficacy index
SE: Spherical equivalent
IOP: Intraocular pressure
ECD: Endothelial cell density
ECV: Endothelial variable coefficient for size
EHE: Endothelial hexagonality
